# The implementation of the functional task exercise programme for elderly people living at home

**DOI:** 10.1186/1471-2474-13-128

**Published:** 2012-07-23

**Authors:** Margot A H Fleuren, Susan Vrijkotte, Marielle P Jans, Renske Pin, Ariette van Hespen, Nico L U van Meeteren, Petra C Siemonsma

**Affiliations:** 1 TNO, Healthy for life, PO Box 2215, 2301 CE, Leiden, The Netherlands; 2 University Medical Center Groningen, Center for Human Movement Sciences, AnthoniusDeusinglaan 1, 9713 AV, Groningen, The Netherlands; 3 Department of Human Physiology & Sports Medicine, VrijeUniversiteit Brussel, Pleinlaan 2, B-1050, Brussels, Belgium; 4 University of Applied Sciences, School of Physiotherapy, Bolognalaan 101, 3584 CJ, Utrecht, The Netherlands; 5 University of the Netherlands Antilles, Faculty of Social and Behavioral Sciences, PO Box 3059, Willemstad, Curaçao

**Keywords:** Elderly, physical exercise, activities of daily living, functional training, determinant analysis, Implementation, Innovations, Health care

## Abstract

**Background:**

The Functional Task Exercise programme is an evidence-based exercise programme for elderly people living at home. It enhances physical capacity with sustainable effects. FTE is provided by physiotherapists and remedial therapists. Although the intervention was found to be effective in a Randomised Controlled Trial, we may not assume that therapists will automatically supply the programme or that elderly people will automatically join the programme. This study protocol focuses on identifying determinants of implementation, developing implementation strategies and studying the effects of the implementation in daily practice.

**Methods/Design:**

Phase 1: The systematic identification of determinants of the implementation of FTE among therapists and the elderly. A questionnaire study was conducted in a random sample of 100 therapists, and interviews took place with 23 therapists and 8 elderly people (aged 66 to 80 years). The determinants were broken down into four categories: the characteristics of the environment, the organisation, the therapists, and the training programme.

Phase 2: Developing and applying strategies adapted to the determinants identified. Fifteen physiotherapists will be trained to provide FTE and to recruit elderly people living at home. The therapists will then deliver the 12-week programme to two groups of elderly, each consisting of six to twelve people aged 70 years or older.

Phase 3: Study of implementation and the impact. To study the actual use of FTE: 1) therapists record information about the selection of participants and how they apply the key features of FTE, 2) the participating elderly will keep an exercise logbook, 3) telephone interviews will take place with the therapists and the elderly and there will be on-site visits. The effects on the elderly people will be studied using: 1) the Patient-Specific Questionnaire, the Timed Up and Go test and a two performance tests. All tests will be performed at the start of the FTE programme, half way through, and at the end of the programme.

**Discussion:**

The number of older people will increase in many countries in the years to come and so the project outcomes will be of interest to policy-makers, insurance companies, health-care professionals and implementation researchers.

## Background

Regular physical activity is recommended, and generally considered to be an important strategy, for the reduction or prevention of functional decline with aging [1]. In addition, physical activity reduces the risk of disease and has a beneficial effect on the impact of a large number of chronic diseases – and the functional consequences – and multimorbidity [2-4]. Despite compelling scientific evidence and recommendations from the government [5] about the required level of daily physical exercise, epidemiological surveys indicate that approximately 20% of older people in the Netherlands can be considered inactive [6]. The number of Dutch people aged 65 and older will increase from 2.3 million in 2005 to approximately 4 million by 2040, more than 25% of the total population [7]. Maintaining well-being and independent living, and preventing functional decline, are important social values. Nevertheless, more than half of Dutch people between 65 and 75, and almost two-thirds of people over 75, have at least one chronic health condition. Furthermore, the majority of older people suffer from impaired functioning or well-being [8]. Old age and disablement are the main determinants of public service utilisation, and especially of health care use [1,9].

Increasing physical activity in the elderly is therefore a relevant and important issue and so we decided to study the implementation of an evidence-based programme for elderly people living at home: Functional Task Exercise (FTE). By contrast with most other exercise programmes for older people, FTE is based on state-of-the-art knowledge about human movement sciences, action theory, motor learning, motivation, rehabilitation medicine, development of frailty and cognitive psychology [10-12]. Previous research looking at older women has shown that, by comparison with the usual care, i.e. strength exercise, FTE is more effective in improving functional performance, and that FTE is the first exercise programme with sustainable effects [11,12]. It is assumed that FTE achieves enduring effects because it enhances older people’s physical capacity and also because it fits in with daily routines. This clearly differentiates FTE from other programmes in regular use. The single-blinded RCT conducted by De Vreede et al. [10,12,13] found an effect size of 1.25, with an average increase of 15%, on the Assessment of Daily Activity (ADAP) test. This is generally considered to be a large effect size [13] and thought to be relevant [11]. Moreover, the FTE programme was very much appreciated by the older participants: they rated it 8.6 on an 11-point scale [14]. Training compliance was also high: an average of 90% of the participants attended all the training sessions [14]. More details about FTE can be found in the methods section.

### Objectives

The objective of the study was threefold. Firstly, to develop an implementation strategy for the introduction of FTE based on the outcomes of an analysis of determinants. Secondly, to apply these strategies and to study the process of implementation in terms of reaching the target population of therapists as well as the elderly. Thirdly, to study the effect of the implementation of the FTE programme in daily practice in terms of physical activity and the physical functioning of the elderly population. An innovation framework was used to structure each step and this has been described in the methods section.

## Methods

### Innovation framework

**Figure 1:**
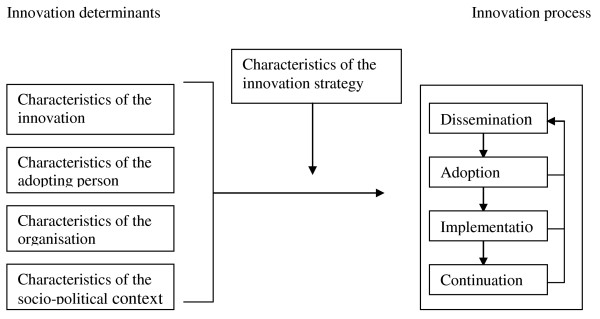
**Framework representing the innovation process and related categories of determinants**[15].

Although FTE has been found to be effective in a RCT, we may not assume that, in daily practice, physiotherapists and remedial therapists will automatically provide FTE or that older people will automatically join an FTE programme. The probability of the actual implementation of innovations like FTE is maximised if they are introduced systematically [15-18]. In the present study, we used a framework based on several theories that has been used in the Netherlands at TNO since 1999 for the introduction and evaluation of innovations in a wide range of domains in Dutch health care [15,19-25]. Figure 1 shows the four main stages in innovation processes. During the dissemination stage every professional must be supplied with the innovation. In the adoption stage, the professional develops positive or negative intentions about using the innovation. In the implementation stage, the professional tries to use the innovation in daily practice and finds out what working with the innovation actually means. In the final stage, the continuation stage, working with the innovation either becomes routine practice or not.

These four main stages in innovation processes can be thought of as success or failure points where the desired change may, or may not, occur. The transition from one stage to the next can be affected, positively or negatively, by various determinants [15,16] (see Figure 1). Determinants can be broken down depending on association with [15]:

1) the innovation (e.g. complexity, relative advantage, compatibility),

2) the adopting person (e.g. skills, outcome expectations, self-efficacy, perceived prevalence of health problem),

3) the organisation (e.g. available expertise, staff turnover, financial recourses, available time),

4) the socio-political context (e.g. patient cooperation, legislation, financial burden on patient).

A detailed understanding of critical determinants is a prerequisite for designing an innovation strategy that can achieve real change. This analysis can be made by assessing which determinants are encountered by both non-users and users when trying to adhere to the innovation [15,23]. If an analysis of determinants is not conducted and/or the applied innovation strategy does not take the relevant determinants into account, the innovation process might fail [15-18]. The failure may be due to focusing on less relevant, or even entirely irrelevant, determinants. Alternatively, the selected strategies/interventions may be inappropriate as a way of steering the relevant determinants of the innovation process. These insights are applied in the present study.

### Intervention: FTE exercise programme

A functional task exercise programme for the elderly (FTE) has been developed and tested [10-12,14]. FTE is a group-based exercise programme to be provided by physiotherapists or remedial therapists (such as Cesar therapists or Mensendieck therapists). It directly targets daily tasks in the domains that are affected early in the aging process. The aim of FTE is to increase the functional independence of older adults in daily life. The programme specifically targets four domains:

1. movements with a vertical component like walking up stairs,

2. movements with a horizontal component like walking around,

3. transporting an object,

4. transfers, for example moving from a lying/sitting/standing position.

During each training session, the participants perform tasks from at least two of the four domains. Each exercise includes three sets of 5–10 repetitions. The 12-week programme is divided into three phases; a practice phase, a variation phase, and a daily tasks phase, therefore increasing the complexity and variability of the exercises step by step. The three phases are described here in brief.

*Practice phase* (2 weeks). The aims of the practice phase are for participants to 1) learn how the exercises are performed, 2) get used to training, and 3) learn how to train at an appropriate intensity. Exercises in this phase consist of short, simple tasks from all four domains: the basic tasks.

*Variation phase* (4 weeks). The aim of the variation phase is to build up the participants’ physical capacities and their ability to use variation and complexity in tasks. In the variation phase, participants apply the basic tasks to various training conditions. Environmental factors are influenced (this involves, for example, distraction using sounds or irregular floors), attributes are used (for example, a shopping bag is used during walking, or something is picked up from the floor), and interaction between participants is encouraged to distract the participants from their tasks.

*Daily tasks phase* (6 weeks). The daily tasks phase consists of a combination of the four domains so that the tasks resemble daily tasks as much as possible. The aim of this phase is to train situations that closely match the participants’ daily activities. Activities are trained beyond the level of variation and complexity that is generally needed for daily activities in order to build up reserve capacity.

During each phase, the therapist can complicate or simplify motor, environment, and cognitive aspects of the tasks in accordance with the abilities of each participant. Each aspect can be changed in a stable or a variable way [10]. For example, the weight or height of an exercise, the number of repetitions, and the difficulty of the exercise can be changed. Alternatively, participants can be asked to count backwards, select objects in a specific order or work together.

### Study design

This study comprised three phases: phase 1, the analysis of determinants (2010); phase 2, the development of implementation strategies, and the recruitment and training of therapists (2011–2012) and phase 3, studying the process and the effect of the implementation (2011–2012). Each phase will be discussed in more detail in subsequent sections. The study design and protocol (number M455) were approved by METOPP medical-ethical committee in Tilburg. No informed consent was required since 1) the study protocol focuses on (identifying determinants of) implementation of the intervention in physiotherapists and remedial therapists, 2) routinely collected data are used to estimate the effects of the implementation in daily practice, and 3) elderly are free to decide to participate in an interview about their expectations or experience of the intervention. The protocol and the data presented in this paper have not been published previously.

### Phase 1: analysis of determinants (2010)

#### Objective

The goal of this phase was to establish an understanding of the critical determinants relating to the adoption and anticipated use (implementation) of FTE by physiotherapists, remedial therapists and the elderly.

#### Participants

Determinants were studied in both therapists and in elderly people living at home. The respondents were physiotherapists or remedial therapists who worked in primary care practices and physiotherapists working in nursing homes. Therapists working in nursing homes were included because they provided their services to elderly people living at home in their vicinity.

First of all, a random sample of 100 therapists registered with the Royal Dutch Society of Physical Therapy (KNGF) were asked to participate in an Internet questionnaire study. The participants consisted of physiotherapists and remedial therapists who were familiar with helping elderly people, or who were interested in training programmes for the elderly.

Secondly, 23 physiotherapists and remedial therapists who were interested in or had experience with FTE were asked to participate in a focus group interview. Former users (n = 2 physical therapists) were interviewed in person. Therapists who had indicated an interest in using FTE in person (n = 10 physical therapists working in primary care, n = 9 physical therapists working in nursing homes, and n = 2 remedial therapists) participated in an online focus group interview. Primary care therapists and nursing home therapists were questioned in separate focus groups, because critical innovation determinants such as finances, available time and appropriate therapy materials might differ between those groups.

Thirdly, eight elderly people living at home (aged 66 to 80) were asked to participate in an individual semi-structured telephone interview. Seven lived independently at home and one lived in the vicinity of an elderly care facility. All were representatives of the FTE target group. Five reported being generally healthy, two said they were not in good health and one reported moderate health. Seven out of eight people had experienced changes in their activities due to aging.

#### Method

The framework for innovation guided the content addressed by the questions. Determinants relating to the context, organisation, adopting person and innovation were studied by progressively building up and detailing information. This was done in therapists using a questionnaire, interviews and three online focus groups. In the elderly, this was done in a semi-structured interview.

First of all, a questionnaire for the therapists was developed with ten questions (requiring yes/no answers) about the adoption and implementation of FTE. For example, one question was whether they were interested in providing FTE for elderly people. Open-ended questions were asked about factors that could encourage or impede the uptake of FTE. The results from the questionnaire were used to verify and refine the interview with former users. In turn, this information was used to optimise the content of the questions for the focus group interviews.

Secondly, online focus groups were scheduled for therapists. The participants received detailed information about the FTE programme beforehand. Three blocks of questions were discussed online over a period of three days. Participants were asked to answer the questions in the morning and to respond to other people’s answers to animate discussion. The forum host asked additional questions to further detail the answers and to encourage discussion. Participants could log on at any time that was convenient given their working schedule. The first block of questions related to the adoption of the FTE programme itself, such as perceived relative advantage, complexity, clearness of procedures, compatibility, outcome expectations or relevance for the elderly. The second block of questions related to determinants that would positively or negatively influence the actual uptake of FTE, such as self-efficacy, knowledge and skills, feasibility, financial burden for elderly people or material resources. All key recommendations/activities in the FTE programme were discussed. Finally, on the last day, critical determinants were discussed in detail and the participants were also asked how they thought the determinants should be integrated in the innovation strategy.

Thirdly, individual interviews took place by telephone with eight elderly people living at home. They received detailed information about the FTE programme beforehand which covered the aims, training frequency and duration, effectiveness, and price. The interview questions were fairly similar to those of the therapist. First, questions were asked about daily physical activities and decreasing activity due to age. Second, the various components of the FTE programme were discussed, as well as the respondents’ opinions about these components. The final questions related to determinants of the actual participation of the respondent in FTE and how elderly people could be encouraged to join the FTE programme.

#### Analysis

For the questionnaires sent to the therapists, frequency tables were drawn up for the answers to the questionnaire to summarise the results. The answers to the interviews and open-ended questions in the questionnaire were independently analysed by two researchers using the four main categories from the innovation framework. The scores were compared and discrepancies were discussed until consensus was achieved. When the data was being coded, it emerged that the respondents often failed to make a clear distinction between determinants relating to the organisation and determinants relating to the socio-political context. The last two categories were therefore combined to produce a broad category “organisational and political context”. This is in line with later publications of Fleuren et al., in which they specify several determinants related to both the level of the organisation and the socio-political context [15,25].

#### Results of the analysis of the determinants

A total of 75 therapists (77%) completed the questionnaire. The majority (88%) were positive about FTE in general. Therapists thought FTE was relevant for the elderly (96%) and for themselves (69%). Eighty-four per cent of the therapists felt FTE was compatible with their working routines and 72% were interested in arranging FTE for the elderly. Fourteen per cent thought training in FTE would be necessary. However, only 11% of the therapists were prepared to pay themselves for the costs of implementing FTE such as costs of FTE training.

The results from the focus group interviews showed that all therapists were enthusiastic about FTE in general. They rated the functionality of the training in particular highly because they were convinced that FTE was closely related to those daily activities that are relevant for elderly people. Therapists also identified effectiveness, and the sustained effects compared with strength exercises, as strengths of the programme.

Although many therapists were willing to adopt FTE, they mentioned several obstacles to actual implementation. Many of the obstacles were related to FTE itself, such as the intensity of the programme (three sessions a week was considered too much), the costs for participants (estimated at € 10 per session) and the over-large group size (up to 12 participants). Determinants linked to the therapists themselves were low self-efficacy in functional training areas, lack of knowledge of the FTE principles, and lack of experience with group training or with training for the elderly. A major obstacle linked to the organisation and social-political context was reimbursement for the therapist for the extra resources required to implement FTE, such as materials, time, staff and education. Related issues were the lack of skills and time to recruit groups of elderly people for FTE. The interviewed therapists were therefore clearly more of the opinion than the therapists in the questionnaire study that training in FTE was needed. This can probably be explained by the more detailed knowledge of the interviewed therapists and the more thorough discussion of what the intervention entails in clinical practice.

In the individual interviews, most elderly people said that FTE would be a good training programme for the elderly. Nevertheless, they thought three training sessions a week was too many, and unrealistic. Two training sessions a week were acceptable to them. Furthermore, they were worried about the costs of FTE. Although most (7 out of 8) elderly people mentioned a decrease in daily activities due to aging, none of them would join the FTE programme for this reason; they seemed to accept a reduction in activity as an inevitable consequence of aging.

#### Product

Phase 1 resulted in an overview of the critical determinants for implementing FTE in daily practice. In summary: 1) therapists’ knowledge of the FTE principles as well as training of the skills required to provide FTE need optimising; 2) therapists’ skills and knowledge relating to the delivery of group training and training for the elderly need to be addressed; 3) therapists’ skills and knowledge relating to ways of contacting and recruiting the appropriate elderly people needs addressing; 4) therapists’ skills and knowledge relating to the delivery of FTE in a financially sustainable way need optimising; 5) FTE can be optimised by taking the critical remarks of the respondents into account, by changing the number of training sessions and the group size for example, and 6) FTE should be presented in a way that appeals to the elderly. These determinants were addressed in the innovation strategies described in Phase 2.

### Phase 2: implementation strategies, recruitment and training of therapists (2011–2012)

#### Objective

The objective of this phase is to develop and apply strategies for the actual implementation and to recruit and train 15 physiotherapists or remedial therapists who will provide FTE to the elderly adults in their primary care practices.

#### Participants

Fifteen physiotherapists and/or remedial therapists will be recruited. Inclusion criteria are:

a) therapists have to be working with elderly people,

b) therapists are willing to implement FTE in their practices,

c) therapists are willing to follow the FTE training,

d) therapists are willing to participate in the study,

e) therapists have graduated and are formally registered with their professional organisations,

#### Method

##### Recruitment

Specialists in the area of geriatric physiotherapy and remedial therapy will be asked to supply contact information for therapists who might be interested in the study.

The professional organisations of geriatric physiotherapists and remedial therapists will be asked to supply addresses of therapists who might be eligible and interested in the study. Furthermore, addresses of eligible therapists will be obtained from the internet and physiotherapists in the TNO network will be contacted. These will mainly be physiotherapists who have participated in previous studies of training for the elderly.

All therapists will receive an e-mail containing global information about the study. Those therapists who express interest will be contacted by phone and will receive detailed information about the FTE programme, the training and the study protocol. Therapists will be contacted until 15 therapists are found who are eligible to participate in this study. Other interested therapists will be placed on a reserve list.

##### Strategy 1: training for therapists

The training for the therapists will consist of three components linked to the determinant analyses in Phase 1: FTE principles and how to provide group training for the elderly, recruitment of elderly, and providing FTE in a financially sustainable way. Each topic is described in more detail below.

a) Training in FTE principles

A critical conclusion of our determinant analysis was that therapists need to learn about the FTE approach. Therapists will therefore be taught the principles underlying FTE. The education module incorporates cooperative learning, and feedback and evaluation. FTE principles will be taught first. This includes the theoretical background, as well as the planning of group progress, documentation of individual progress and the use of clinimetric measures to document changes and plan progress. Secondly, the use of FTE will be demonstrated by teachers with 10 or more years of experienced. Thirdly, the therapists will practise FTE skills and using FTE themselves. The therapists will receive feedback from the teachers about their performance. During the training, the therapists will also be made aware of the importance of giving their participants a range of options to remain active after the FTE programme such as joining social, hobby or activity groups that match the personal needs of the participants and encourage them to stay active.

The determinant analysis showed that it was necessary to train knowledge and skills relating to the target population of elderly people because specific skills are required when addressing the problems faced by older people. The training will therefore focus on social, communicational, motivational and safety issues. Therapists will also learn how to provide group training for the elderly. In line with the determinant analysis, the FTE programme will be adapted to the needs of the participants and the circumstances of the therapists’ local settings. However, key activities and the FTE principles in the programme will not be changed.

b) Recruitment of elderly participants

Therapists will receive training and guidance in building community networks for contacting and recruiting the elderly for FTE. Therapists will be made aware of the different ways of contacting the elderly: through GPs, home care, the community, private care, media and so on. The therapists will learn how to develop a network strategy that is adapted to their local situation. After the training, the network strategy will be immediately applicable and ready to use.

c) Business case

It is important for the FTE programme to be delivered in a way that is financially sustainable for both therapists and the elderly. Financial sustainability was found to be a critical determinant reported by the elderly and the therapists: the programme will not be provided routinely if the costs are too high. The therapists will learn how to draw up a business case (a detailed plan of how to implement FTE, including the expected time, costs, financing, and risks) specifically for their own practice. This means that the business case will be immediately applicable and ready to use during and after the training.

Four teachers will be responsible for the training. Each teacher has his/her own specialty, i.e. FTE principles and application, building community networks, business case development, and group training for elderly. The therapists will receive 20 hours of training over three days. Homework assignments relating to each topic covered by the course will be given and the assignments will be discussed on the next day of training. Therapists will spend an average of 20 hours on homework assignments.

Meetings will be at two-week intervals so that therapists have enough time for the assignments and to familiarise themselves with the FTE principles. At the same time, an online forum will be available for asking questions and sharing information. Therapists will receive support during the implementation process to enhance the likelihood of successful implementation.

##### Strategy 2: adaptation of FTE

The intensity of the programme (number of sessions per week) and the group size will be adapted in the light of the critical remarks that emerged from the determinant analysis. As the determinant analysis showed, the elderly will not automatically see themselves as the target population for FTE. To further acceptance, the FTE programme will be presented to the elderly as an attractive programme.

#### Analyses

To complete the training, each therapist will be given an individual portfolio containing all homework assignments. The training will be evaluated using questionnaires completed immediately after the training and after the therapists have delivered FTE to elderly people for the first time (see Phase 3).

#### Product

The products of this phase are 1) fifteen physiotherapists and remedial therapists trained to implement and execute the FTE programme in their practices, 2) a feasible programme that is adjusted to the needs of therapists and elderly, 3) sound business cases and community network strategies for each local setting.

### Phase 3: study of the effect of FTE (2011–2012)

#### Objective

The aim of the final stage of this study is twofold; 1) to study the process of implementation in terms of reaching the target population of the physiotherapists or remedial therapists as well as the intended proportion of the elderly population, and 2) to study the implementation, and the effect of the implementation, of the FTE programme in daily practice with regard to physical activity and physical functioning of elderly people (as measured with self-report and physical performance measures).

#### Participants

Each physiotherapist or remedial therapist who completes the training course will deliver the FTE programme to two groups of elderly people. The second group will start when the first group has finished the 12-week FTE programme. To safeguard against possible therapist drop-out, one extra therapist will be trained. Each group consists of six to twelve elderly people aged 70 years or older who have slight problems with activities of daily living. The targeted elderly people will live independently and be willing to follow FTE to improve their independence and their activities of daily living. They will also have to be able to exercise strenuously without being limited by comorbidities or other disabilities.

#### Method

##### Evaluation of process of implementation

The therapists will keep records about how they recruit elderly people and of the reasons for participation/non-participation of the elderly. Furthermore, during the 12-week FTE programme, the therapists will keep records of the number of participants who are present and the reasons for any absence.

##### Evaluation of the use of FTE

Determinants of use. Before and after the FTE programme, all therapists will receive a questionnaire looking at the determinants of the use of FTE, as well as intentions about the continued use of FTE.

Actual use. The actual use, and the quality of use, of the key activities in the FTE programme will be assessed in three ways: 1) the therapists will complete a record form for each group, detailing the selection of participants, how well they managed to apply the key features of FTE, and what obstacles and facilitating factors were encountered, 2) the participating elderly will keep a logbook of exercises for each training session in which they will note the exercises, the variations that were used, the number of repetitions, the weight used, the perceived intensity of the exercise, and the time needed to perform an exercise, 3) telephone interviews (with therapists and elderly) and site visits will take place in which the researchers will verify the implementation of the FTE, implementation problems and local solutions.

The use of several sources of information will make it possible to study the use of FTE, as well as changes in use resulting from, for example, increased therapist experience with FTE. Furthermore, the completeness of use – the number of activities performed – and the quality of use will be studied.

##### Effects of the adjusted FTE

Therapists will recruit the elderly and train them using the FTE programme. Before the start of the FTE programme, there will be an intake procedure to check for comorbidities and other limiting factors. During the intake and at the start of the FTE programme, the following questionnaire and tests will be used:

the Patient-Specific Questionnaire (PSQ – in Dutch, the “PatientSpecifiekeKlachtenlijst”) measures the functional status of a patient with respect to the three to five most important physical complaints [26];

the Timed Up and Go test (TUG) measures the basic mobility of older people [27];

the 10 meter walking test measures the walking speed;

a patient-specific performance test (related to the goals of the patient as determined by the PSQ). This test can be different for each participant since it is related to their specific, personal physical problem.

The PSQ questionnaire and the tests will be repeated halfway and at the end of the FTE programme in order to document changes and to provide the therapist with additional information about how to adjust training intensity.

#### Analysis

Qualitative analyses will be used to evaluate the implementation strategy. ANOVA for repeated measures will be used to determine the effect of the FTE programme.

#### Product

The effects of the adjusted FTE programme and the adjustments will be documented and made available as products. After the completion of the final phase of the study, the adjustments needed to adapt the FTE programme in daily practices will be known, as will the training effects of the adapted FTE programme. It will also become clear which determinants have affected the outcome of the study. Finally, the study will provide input for a national implementation strategy based on the experiences of this smaller implementation study.

## Discussion

The overall aim of this implementation study is to facilitate the nationwide implementation of a Functional Training Exercise (FTE) programme. Maintaining well-being and independent living, and preventing functional decline in elderly people living at home, are important values in Dutch society. FTE is the first exercise programme for the elderly that achieves sustainable improvements in performance and in the prevention of functional decline. As in many countries, the number of elderly people will increase over the next 25 years in the Netherlands and so the project outcomes will be of interest to policy-makers, insurance companies and health-care professionals, as well as implementation researchers and implementation consultants/advisors.

In our opinion the biggest difficulty with the study will be the recruitment of both therapists and elderly participants. Concerns about therapists include the recruitment of therapists with a continued interest in the FTE programme. Ideally, therapists will be actively involved in the training and homework, implement the FTE in their practice, provide FTE to at least two groups of participants and continue to provide the programme after the end of the study. Furthermore, time and money constraints are a potential threat to actual participation in the study. Another problem is recruiting participants within the time window of the study. Our control of both issues is limited.

## Competing interests

The authors declare that they have no competing interests.

## Authors’ contributions

All authors have contributed equally to the design of the study and the writing of this paper. MF led the writing process. All authors have commented on drafts and approved the final version. MJ, MF, SV, RP and PS conducted the determinant analysis (Phase 1).

## Pre-publication history

The pre-publication history for this paper can be accessed here:

http://www.biomedcentral.com/1471-2474/13/128/prepub
